# miR-29s function as tumor suppressors in gliomas by targeting TRAF4 and predict patient prognosis

**DOI:** 10.1038/s41419-018-1092-x

**Published:** 2018-10-22

**Authors:** Cuijuan Shi, Chun Rao, Cuiyun Sun, Lin Yu, Xuexia Zhou, Dan Hua, Run Wang, Wenjun Luo, Zhendong Jiang, Junhu Zhou, Qian Wang, Shizhu Yu

**Affiliations:** 1 Department of Neuropathology, Tianjin Neurological Institute, Tianjin Medical University General Hospital, Tianjin, 300052 China; 2 Tianjin Key Laboratory of Injuries, Variations and Regeneration of the Nervous System, Tianjin, 300052 China; 3 Key Laboratory of Post-trauma Neuro-Repair and Regeneration in Central Nervous System, Ministry of Education, Tianjin, 300052 China; 4 Department of Biochemistry and Molecular Biology, School of Basic Medical Sciences of Tianjin Medical University, Tianjin, 300070 China; 5 Lab of Neuro-Oncology, Tianjin Neurological Institute, Tianjin Medical University General Hospital, Tianjin, 300052 China

## Abstract

Robust proliferation and apoptosis inhibition of tumor cells are responsible for the high mortality and poor outcome of patients with high-grade gliomas. miR-29a/b/c have been reported to be important suppressors in several human tumor types. However, their exact roles in gliomagenesis and their relevance to patient prognosis remain unclear. In this study, using 187 human glioma specimens and 20 nontumoral brain tissues, we demonstrated that the expression of miR-29a/b/c decreased progressively as the grade of glioma and the Ki-67 index increased. However, the expression of TRAF4, the functional target of miR-29a/b/c, exhibited the inverse trend, and its level was inversely correlated with the levels of miR-29a/b/c. A Kaplan–Meier analysis demonstrated that the miR-29a/b/c and TRAF4 levels were closely associated with patient survival even in patients with the same tumor grade and identical *IDH* gene status. A functional study verified that miR-29a/b/c induced apoptosis and suppressed the proliferation of glioma cells by directly targeting TRAF4. An investigation of the mechanism revealed that miR-29a/b/c promoted apoptosis through the TRAF4/AKT/MDM2 pathway in a p53-dependent manner, while miR-29a/b/c induced G1 arrest and inhibited tumor cell proliferation by blocking the phosphorylation of AKT and GSK-3β, and the expression of cyclin D1 and c-Myc. Furthermore, TRAF4-knockdown perfectly simulated the anti-glioma effects of miR-29a/b/c. These findings enrich our understanding of gliomagenesis, highlight the prognostic value of miR-29a/b/c and TRAF4, and imply their potential therapeutic roles in malignant gliomas.

## Introduction

Gliomas are the most frequent primary brain tumors in adults^1,2^, and malignant gliomas, especially glioblastomas, are aggressive and lethal neoplasms characterized by rapid growth and persistent infiltration, which means that a radical resection is almost impossible^3^. Although unlimited proliferation and inhibition of tumor cell apoptosis have been accepted as the key reasons for the rapid growth of malignant gliomas, the underlying epigenetic and genetic changes are still poorly understood^4^. Moreover, prognostic biomarkers and therapeutic targets for gliomas have not been fully characterized. In addition, a thorough study on the molecular mechanisms of glioma formation and malignant progression is a prerequisite for the screening of valuable diagnostic and prognostic biomarkers and for the optimization of the therapeutic strategies against malignant gliomas.

Recent studies have demonstrated that miRNAs are important epigenetic regulators in tumorigenesis and promising biomarkers for prognosis^5–10^. The human miR-29 family consists of three closely related members, miR-29a, b, and c^11^. The abnormal decrease in their levels and the association of that decrease with a poor prognosis have been reported in various malignancies^12–20^. Moreover, recent efforts on the manipulation of exogenous miR-29 family members represent appealing approaches to anti-tumor therapy^17,21^, which suggests that miR-29a/b/c can be used as prognostic biomarkers and that they are therapeutic candidates for these tumors. However, their prognostic relevance and tumor suppressive effects still need to be fully elucidated in gliomas.

Tumor necrosis factor receptor-associated factor-4 (TRAF4), which is a cytoplasmic adaptor that functions as an E3 ubiquitin ligase, has been shown to be overexpressed in several malignancies and to participate in tumorigenic processes^22–24^. Two previous studies have reported that TRAF4 was a natural target of miR-29 in metastatic prostate cancer and human fetal lung fibroblast IMR-90 cells^24,25^. However, to the best of our knowledge, the expression pattern of TRAF4 in gliomas and its exact roles in gliomagenesis remain largely elusive.

In the present study, we demonstrated that miR-29a/b/c induced glioma cell apoptosis through the TRAF4/AKT/MDM2 pathway in a p53-dependent manner, and restrained cell proliferation by directly targeting the TRAF4/AKT/GSK-3β pathway. Our results also implied the potential value of miR-29a/b/c and TRAF4 in the prognosis of glioma patients and as potential therapies for malignant gliomas.

## Materials and methods

### Tissue samples and clinical data

In all, 187 surgical specimens of human astrocytic gliomas and 20 nontumoral brain tissues were collected form Tianjin Medical University General Hospital (TMUGH) and were included in the present study after the patients provided written consent. The specimens were fixed in 3.7% buffered formaldehyde immediately after surgical excision and were embedded in paraffin (FFPE samples). Then, 5-μm-thick serial tissue sections were prepared for hematoxylin and eosin staining, miR-29a/b/c in situ hybridization, and immunohistochemistry (IHC) for TRAF4 and Ki-67. The pathologic diagnoses were rendered independently by two neuropathologists according to the 2016 World Health Organization (WHO) classification of central nervous system tumors^1^. The clinicopathologic features, including the WHO grades, *IDH1/2* gene statuses, and KPS scores, are summarized in Supplementary Table 1. All 187 glioma patients with complete clinical information were followed-up from the date of operation until 31 December 2014; the follow-up time ranged from 3.6 to 88.3 months.

Independent RNA-seq data of 638 human glioma samples were obtained from the Cancer Genome Atlas (TCGA) database (https://cancergenome.nih.gov/). The expression levels of TRAF4 were measured using the Illumina HiSeq RNA Sequencing platform. After log_2_ transformation, the expression data were subjected to a Kaplan–Meier analysis to verify the relationships between the TRAF4 levels and the overall survival (OS) (638 cases) and disease-free survival (DFS) (501 cases) of the glioma patients. For Oncomine data analyses (http://www.oncomine.org), the database was searched for TRAF4 using the following filter setting: Cancer vs. Normal analysis in brain and CNS cancer, *P*-value of <1 × 10^−^^4^, and gene rank in the top 10%. The values from each published data set were linked to the graphical representations of the original data. TRAF4 mRNA levels were compared between human glioblastoma tissues and normal brain tissues (control) by Student’s *t*-test.

### In situ hybridization and IHC

In situ hybridization (ISH) and IHC were performed as previously described^6,7^. For ISH, the locked nucleic acid-modified and digoxin-labeled probes for miR-29a/b/c and the control oligonucleotide with scrambled sequence (Scr) were purchased from TaKaRa (Dalian, China; Supplementary Table 2). The rhodamine (TRITC)-conjugated anti-digoxin antibody and 4′,6-diamidino-2-phenylindole were purchased form Roche (Indianapolis, IN, USA). IHC staining was performed with primary antibodies including a rabbit anti-human TRAF4 antibody (Santa Cruz Biotechnology, Santa Cruz, CA, USA) and a rabbit anti-human Ki-67 antibody (Millipore, Billerica, MA, USA). ISH and IHC images were acquired with a DM6000B fluorescence microscope (Leica, Wetzlar, Germany). For each slice, the number of cells in six randomly selected ×400 microscopic fields was counted with Image-Pro Plus 5.0 (Leica, Wetzlar, Germany), and the labeling index (LI %) was calculated as the proportion of positive cells.

### Cell cultures, lentiviruses, and stable sub-cell lines

The human glioblastoma cell lines U87MG and DBTRG-05MG were obtained from the American Type Culture Collection (ATCC, Manassas, VA, USA), while the SNB19, U251, and LN308 cell lines were purchased from the China Academia Sinica Cell Repository (Shanghai, China). The TJ905 cell line, which was derived from a Chinese patient with glioblastoma, was established and maintained by our lab. The immortalized human astrocyte cell line UC2 was used as the nontumoral control. The DBTRG-05MG cell line was maintained in RPMI-1640 (Gibco, Grand Island, NY, USA), while the others were maintained in Dulbecco's modified Eagle's medium (Gibco, Grand Island, NY, USA). All the media were supplemented with 10% fetal bovine serum (Gibco, Grand Island, NY, USA), and the cells were cultured at 37 °C in a humidified atmosphere containing 5% CO_2_. The recombinant lentiviruses expressing miR-29a, miR-29b, miR-29c, or the scrambled control (Scr) were constructed and packaged by Genechem (Shanghai, China). The corresponding sub-cell lines of U87MG, DBTRG-05MG, SNB19, and U251 were established by lentivirus infection and puromycin selection. The expression efficiencies of the exogenous miRNAs were quantified by quantitative reverse transcriptase-PCR (qRT-PCR).

### Oligonucleotides, plasmids, and cell transfection

U87MG and SNB19 cells were transfected with either the siRNAs targeting TRAF4 (si-TRAF4#1 or si-TRAF4#2) or the scrambled control oligonucleotide (Scr; RiboBio, Guangzhou, China; Supplementary Table 3) using X-tremeGENE siRNA Transfection Reagent (Roche, Indianapolis, IN, USA). The TRAF4 expression plasmid p-TRAF4 was constructed by GeneCopoeia (Rockville, MD, USA) and was validated by DNA sequencing. Plasmid transfection was performed using X-tremeGENE HP DNA Transfection Reagent (Roche, Indianapolis, IN, USA).

### Quantitative RT-PCR

qRT-PCR was performed as previously described^7^. The Bulge-Loop™ miRNA qRT-PCR primer sets for hsa-miR-29a/b/c were purchased from Ribobio (Guangzhou, China). Total RNA (1 μg) was reverse-transcribed with either the miRNA-specific primers or an Oligo dT primer using a Reverse Transcription System Kit (Promega, Fitchburg, WI, USA). Subsequently, qRT-PCR was performed with a GoTaq qPCR Master Mix Kit (Promega, Fitchburg, WI, USA) to quantify the miRNA and mRNA levels using U6 and GAPDH as the internal controls, respectively. The specific primers used for TRAF4 mRNA detection are listed in Supplementary Table 4. The fold changes of the miRNA and mRNA levels were calculated by the 2^−ΔΔCt^ method.

### Flow cytometry assay

For the apoptosis assay, cells were labeled with an Annexin V-APC/PI Apoptosis Detection Kit (KeyGEN, Nanjing, China) according to the manufacturer’s instructions. For cell cycle analysis, cells were harvested, washed with ice-cold PBS, fixed in 70% ethanol at 4 °C overnight, and labeled with PI (Solarbio, Beijing, China) in the presence of RNase A (Solarbio, Beijing, China) at 37 °C for 30 min. Then, the cells were read on an Accuri C6/FACSCalibur Flow Cytometer (BD Biosciences, Franklin Lakes, NJ, USA), and the data were processed with FlowJo/ModFit LT software.

### Cell apoptosis assays

Alkaline single cell gel electrophoresis (SCGE) detection was performed as previously described^7^. Briefly, cells (1 × 10^4^) were harvested and suspended in 0.5% (w/v) low melting agarose at 37 °C, and then the suspensions were layered on slides pre-coated with a layer of 0.6% (w/v) normal-melting agarose. The slides were then immersed in cold lysing solution for 2 h at 4 °C. After electrophoresis, the slides were stained with ethidium bromide, and the images were acquired using a DM6000B fluorescence microscope (Leica, Wetzlar, Germany). Apoptotic indexes [AIs(%)] were calculated as apoptotic cell numbers/total cell numbers × 100%. Caspase 3/7 activity was measured with a Caspase-Glo® 3/7 Assay Kit (Promega, Fitchburg, WI, USA) according to the manufacturer’s instructions. Luminescence was measured after a 30-min incubation with Caspase-Glo® 3/7 Reagent in a Synergy™ 2 Multi-Mode Microplate Reader (BioTek Instruments, Winooski, VT, USA).

### Cell proliferation assays

A 5-ethynyl-2′-deoxyuridine (EdU) incorporation assay was performed with a Cell-Light^TM^ EdU Apollo^®^ 567 In Vitro Imaging kit (RiboBio, Guangzhou, China). Cells (1.5 × 10^3^ cells/well) were seeded in 96-well plates and incubated with EdU (50 µM) for 2 h. The cells were then fixed in 4% paraformaldehyde and stained with Apollo and Hoechst 33342. Cells in five randomly selected microscopic fields were counted and the images were captured with a fluorescence microscope (Olympus, Tokyo, Japan). EdU-positive rates (%) were calculated as EdU-positive cell numbers/Hoechst-stained cell numbers × 100%. For the MTS assay, cells (1.5 × 10^3^ cells/well) were seeded in 96-well plates and incubated for 24–120 h. Before measurements were obtained, the supernatants were replaced with fresh medium containing 3-(4,5-dimethylthiazol-2-yl)-5-(3-carboxymethoxyphenyl)-2-(4-sulfophenyl)-2H-tetrazolium (MTS, Promega, Fitchburg, WI, USA), and the cells were cultured for another 2.5 h. Then the absorbance at 490 nm was measured with a Synergy™ 2 Multi-Mode Microplate Reader (BioTek Instruments, Winooski, VT, USA). Data at each interval during a 24-h period were documented.

### Target prediction and dual-luciferase reporter assay

The candidate targets of miR-29a/b/c were predicted by Targetscan, PicTar, and MicroCosm. The DNA fragment corresponding to the 3′-untranslated region of TRAF4 mRNA (TRAF4-3′-UTR-WT) was amplified by RT-PCR and inserted into the pEZX-MT01 vector (GeneCopoeia, Rockville, MD, USA). Then, target region 1 or 2 of miR-29a/b/c was deleted individually (TRAF4-3′-UTR-MT1 and TRAF4-3′-UTR-MT2) by site-directed mutagenesis PCR using the primers listed in Supplementary Table 5. The recombinant luciferase reporter plasmids containing TRAF4-3′-UTR-WT, TRAF4-3′-UTR-MT1, and TRAF4-3′-UTR-MT2 were named p-WT, p-MT1, and p-MT2, respectively. Firefly and Renilla luciferase activities were detected with a Dual-Luciferase Reporter Assay System (Promega, Fitchburg, WI, USA) according to the manufacturer’s instructions in a Synergy™ 2 Multi-Mode Microplate Reader (BioTek Instruments, Winooski, VT, USA). Firefly luciferase activity was adjusted according to that of Renilla luciferase to normalize the differences in transfection efficiencies.

### Western blotting

Western blotting was performed as described previously^6^. Rabbit anti-human TRAF4 antibody and mouse anti-human MDM2 and p53 antibodies were purchased from Santa Cruz Biotechnology (Santa Cruz, CA, USA). Rabbit anti-human p-AKT (phospho S473), AKT, p-MDM2 (Phospho Ser166), Bax, p-GSK-3β (phospho S9), GSK-3β, cyclin D1, and c-Myc antibodies were purchased from CST (Boston, MA, USA). Mouse anti-human β-actin and GAPDH antibodies were purchased from Boster (Wuhan, China).

### Statistical analysis

Data are presented as the means ± standard deviation (SD). Corresponding data in this study were analyzed by one-way ANOVA, *χ*^2^ test, Pearson correlation analysis, Kaplan–Meier analysis, log-rank test, and Student’s *t*-test. In the survival analysis, the median of each glioma cohort was used as the cutoff for risk stratification. All the analyses were performed using the SPSS 21.0 software package (IBM, Chicago, IL, USA), and statistical significance was assigned at *P* *<* 0.05 (*/^▲^), *P* *<* 0.01 (**/^▲▲^), or *P* *<* 0.001 (***/^▲▲▲^). All the experiments on cell lines were performed at least three times with triplicate samples.

## Results

### miR-29a/b/c are decreased in human gliomas and correlate with poor prognosis

**Fig. 1:**
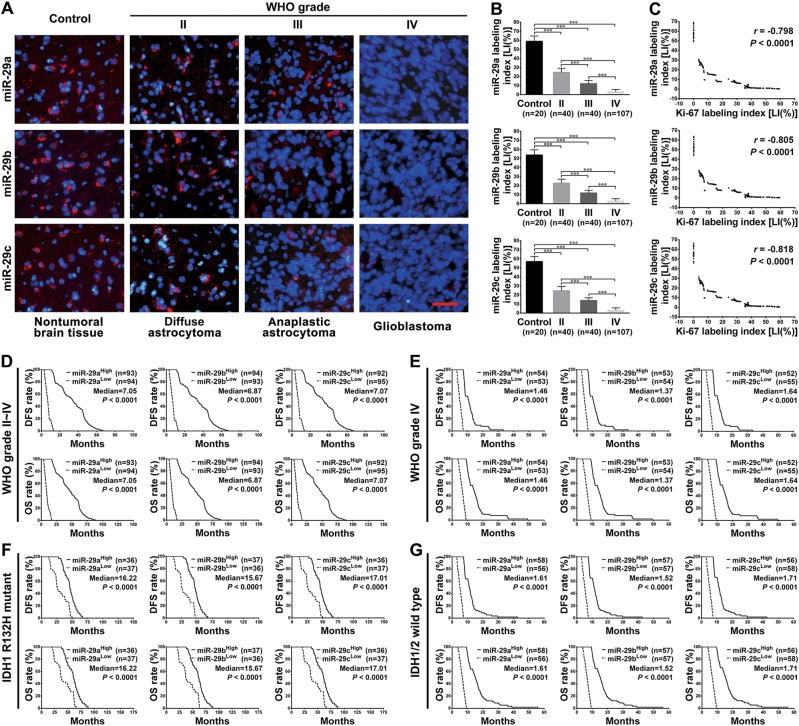
miR-29a/b/c levels are correlated with glioma grade, tumor cell proliferation, and patient prognoses. **a** Representative images of miR-29a/b/c in situ hybridization. Perinuclear miR-29a/b/c were labeled with TRITC (red) while cell nuclei were counter-stained with DAPI (blue). Scale bar: 50 μm. **b** Comparisons of the labeling indexes [LIs(%)] of miR-29a (top), miR-29b (middle), and miR-29c (bottom) among the control and the glioma groups. The LIs (%) of each sample were calculated according to the percent ratio of positive cells to total cells. Data are presented as the mean ± SD. ***P *<* 0.001. **c** A Pearson correlation analysis shows that the LIs of miR-29a (top), miR-29b (middle), and miR-29c (bottom) are all inversely correlated with the Ki-67 index. **d–g** Kaplan–Meier survival analyses of the associations between miR-29a (left), miR-29b (middle) or miR-29c (right) and the DFS (upper) and OS (lower) of the patients. The analyses were conducted in the cohort of all glioma patients (**d**), patients with grade IV gliomas (**e**), patients with IDH1 R132H mutant gliomas (**f**), and patients with IDH1/2 wild-type gliomas (**g**). Patients were stratified into high and low expression subgroups using the medians of the miR-29a/b/c LIs of the corresponding cohort

To reveal the relationship among miR-29a/b/c levels and glioma grade, tumor cell proliferation and patient prognoses, we detected the endogenous levels of miR-29a/b/c and Ki-67 in 187 human glioma specimens and 20 nontumoral control brain tissues. We found that miR-29a/b/c mainly located at the perinuclear zone and their levels were significantly lower in gliomas than in control brain tissues (*P* < 0.001) and that the levels declined progressively as the glioma grade increased (*P* *<* 0.001; Fig. 1a, b). A Pearson analysis showed that all the labeling indexes of the three miR-29s were inversely correlated with the proliferation index (Ki-67 LI; miR-29a: *r* = −0.798; miR-29b: *r* = −0.805; miR-29c: *r* = −0.818; *P* *<* 0.0001; Fig. 1c and Supplementary Fig. 1a, b). Importantly, the decreases in miR-29a/b/c levels were significantly associated with the wild-type *IDH1/2* gene, older age, advanced tumor grade, and higher Ki-67 LI (*P* *<* 0.0001; Table 1). The qRT-PCR results verified that the miR-29a/b/c expression levels were also significantly reduced in six human glioblastoma cell lines compared with the human astrocyte cell line UC2 (Supplementary Fig. 2a, b). Kaplan–Meier analyses demonstrated that the patients with relatively higher miR-29a/b/c levels exhibited longer DFS (*P* < 0.0001) and OS (*P* < 0.0001; Fig. 1d), even within the cohort of patients with the same tumor grade (Fig. 1e and Supplementary Fig. 3a, b), identical IDH status (Fig. 1f, g), similar age, and KPS (Supplementary Fig. 4a–d). These data demonstrate the inverse association of miR-29a/b/c levels with glioma grade and tumor cell proliferation, and imply their potential value as prognostic biomarkers for glioma patients.

**Table 1:** Correlation between miR-29a/b/c and TRAF4 expressions and clinicopathological characteristics of 187 glioma patients

Feature	Number of cases	MiR-29a LI		*χ*^2^*/P*-value		MiR-29b LI		*χ*^2^*/P*-value	MiR-29c LI		*χ*^2^*/P*-value	TRAF4 LI		*χ*^2^*/P*-value
		High	Low			High	Low		High	Low		High	Low	
**IDH status**														
Mutant type (IDH1 R132H)	73	73	0	*χ*^2^ = 121.033 *P* < 0.0001	73		0	*χ*^2^ = 118.472 *P* < 0.0001	73	0	*χ*^2^ = 123.650 *P* < 0.0001	7	66	*χ*^2^ = 77.191 *P* < 0.0001
Wild type (IDH1/2)	114	20	94		21		93		19	95		86	28	
**Gender**														
Male	114	50	64	*χ*^2^ = 4.029 *P* = 0.045		52	62	*χ*^2^ = 2.529 *P* = 0.112	50	64	*χ*^2^ = 3.330 *P* = 0.068	61	53	*χ*^2^ = 1.666 *P* = 0.197
Female	73	43	30			42	31		42	31		32	41	
**Age**														
Age < 50	81	60	21	*χ*^2^ = 33.868 *P* < 0.0001		62	19	*χ*^2^ = 39.464 *P* < 0.0001	61	20	*χ*^2^ = 38.979 *P* < 0.0001	22	59	*χ*^2^ = 29.123 *P* < 0.0001
Age ≥ 50	106	33	73			32	74		31	75		71	35	
**KPS**														
KPS < 90	117	50	67	*χ*^2^ = 6.122 *P* = 0.013		50	67	*χ*^2^ = 7.094 *P* = 0.008	49	68	*χ*^2^ = 6.696 *P* = 0.010	63	54	*χ*^2^ = 2.116 *P* = 0.146
KPS ≥ 90	70	43	27			44	26		43	27		30	40	
**WHO grade**														
II	40	40	0	*χ*^2^ = 141.316 *P* < 0.0001		40	0	*χ*^2^ = 138.326 *P* < 0.0001	40	0	*χ*^2^ = 144.372 *P* < 0.0001	0	40	*χ*^2^ = 74.668 *P* < 0.0001
III	40	40	0			40	0		40	0		12	28	
IV	107	13	94			14	93		12	95		81	26	
**Ki-67 LI**														
LI < 29.01	93	91	2	*χ*^2^ = 171.342 *P* < 0.0001		93	0	*χ*^2^ = 183.042 *P* < 0.0001	91	2	*χ*^2^ = 175.212 *P* < 0.0001	13	80	*χ*^2^ = 94.607 *P* < 0.0001
LI ≥ 29.01	94	2	92			1	93		1	93		80	14	
**Predominant side**														
Left	95	44	51	*χ*^2^ = 1.201 *P* = 0.549		43	52	*χ*^2^ = 2.109 *P* = 0.348	43	52	*χ*^2^ = 1.495 *P* = 0.473	50	45	*χ*^2^ = 0.686 *P* = 0.709
Right	84	44	40			46	38		44	40		39	45	
Middle	8	5	3			5	3		5	3		4	4	
**Predominant location**														
Frontal lobe	103	65	38	*χ*^2^ = 24.340 *P* = 0.001		64	39	*χ*^2^ = 20.071 *P* = 0.005	64	39	*χ*^2^ = 23.292 *P* = 0.002	40	63	*χ*^2^ = 18.927 *P* = 0.008
Temporal lobe	54	15	39			17	37		15	39		34	20	
Parietal lobe	15	6	9			6	9		6	9		10	5	
Occipital lobe	8	2	6			2	6		2	6		7	1	
Insular lobe	1	1	0			1	0		1	0		0	1	
Cerebellum	4	3	1			3	1		3	1		1	3	
CPA	1	0	1			0	1		0	1		0	1	
Third ventricle	1	1	0			1	0		1	0		1	0	

*LI* labeling index, *KPS* Karnofsky performance score

### miR-29a/b/c induce apoptosis and suppress the proliferation of glioma cells

**Fig. 2:**
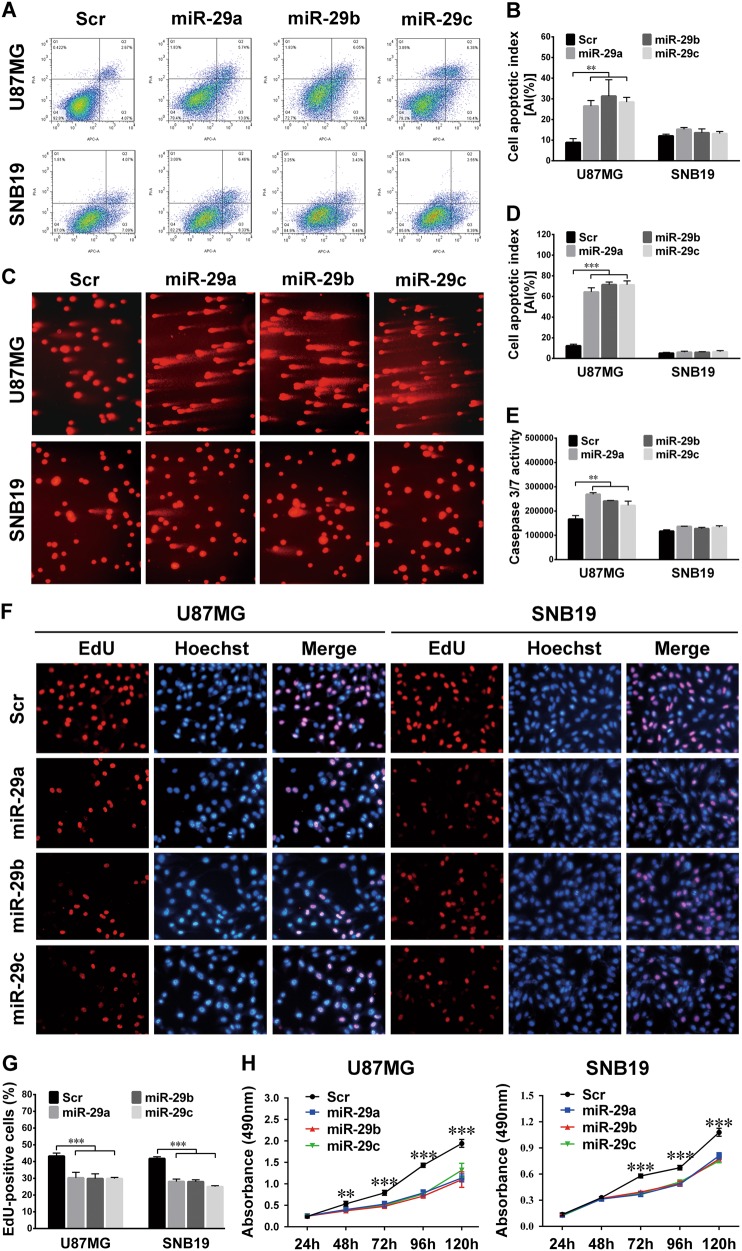
miR-29a/b/c induce apoptosis and inhibit the proliferation of glioma cells. **a, b** The representative FCM cell apoptosis analysis results (**a**) and the apoptotic indexes (AIs%; **b**). The AIs% were compared among the miR-29a/b/c-overexpressing sub-cell lines and the control sub-cell lines (Scr) of U87MG and SNB19 cells. **c**, **d** Representative results of SCGE (**c**), AIs were calculated as the percent ratio of long-tailed cells to total cells (**d**) and compared as described in **b**. **e** Results of the caspase 3/7 activity analysis of the cells as indicated. **f, g** Representative images of the EdU assay (**f**) and the quantification results (**g**). The EdU-positive and Hoechst-stained cells were counted in the microscopic fields at ×200. **h** Growth curves of the above sub-cell lines. The absorbances at 490 nm at the indicated time points were measured by MTS assays. All the experiments were performed at least in triplicate and the data in **b**, **d**, **e**, **g**, and **h** are presented as the mean ± SD. **P < 0.01; ***P < 0.001

To investigate the effects of miR-29a/b/c on tumor cell apoptosis and proliferation, we established sub-cell lines that stably overexpressed miR-29a/b/c (termed miR-29a, miR-29b, and miR-29c) or the control (Scr). The expression efficiencies of the miRNAs were verified by qRT-PCR (*P* < 0.01–0.001; Supplementary Fig. 5a, b). For U87MG with the wild-type *TP53* gene^26,27^, the apoptosis indexes (AIs) of the miR-29a/b/c sub-cell lines were significantly higher than those of the Scr, as indicated by the results of flow cytometry (FCM) (*P* *<* 0.01; Fig. 2a, b), SCGE (*P* *<* 0.001; Fig. 2c, d), and the caspase 3/7 activity assay (*P* *<* 0.01; Fig. 2e). These results were also obtained in DBTRG-05MG cells in which the *TP53* gene was also wild type^28^ (*P* *<* 0.001; Supplementary Fig. 5c, d). However, in SNB19 and U251 cells, which bear a mutation at amino acid 273 of p53 ^27,29,30^, no obvious difference in apoptosis was observed between the control and the miR-29a/b/c-overexpressing groups (Fig. 2a–e and Supplementary Fig. 5c, d). The EdU and MTS assays showed that miR-29a/b/c could effectively inhibit the proliferation of U87MG and SNB19 cells (*P* *<* 0.01–0.001; Fig. 2f–h). Combining with the above findings that miR-29a/b/c LIs were inversely correlated with the Ki-67 LI, our results suggest that, as pro-apoptotic and anti-proliferative miRNAs, miR-29a/b/c are effective glioma suppressors.

### TRAF4 is a direct target of miR-29a/b/c in human glioma cells

**Fig. 3:**
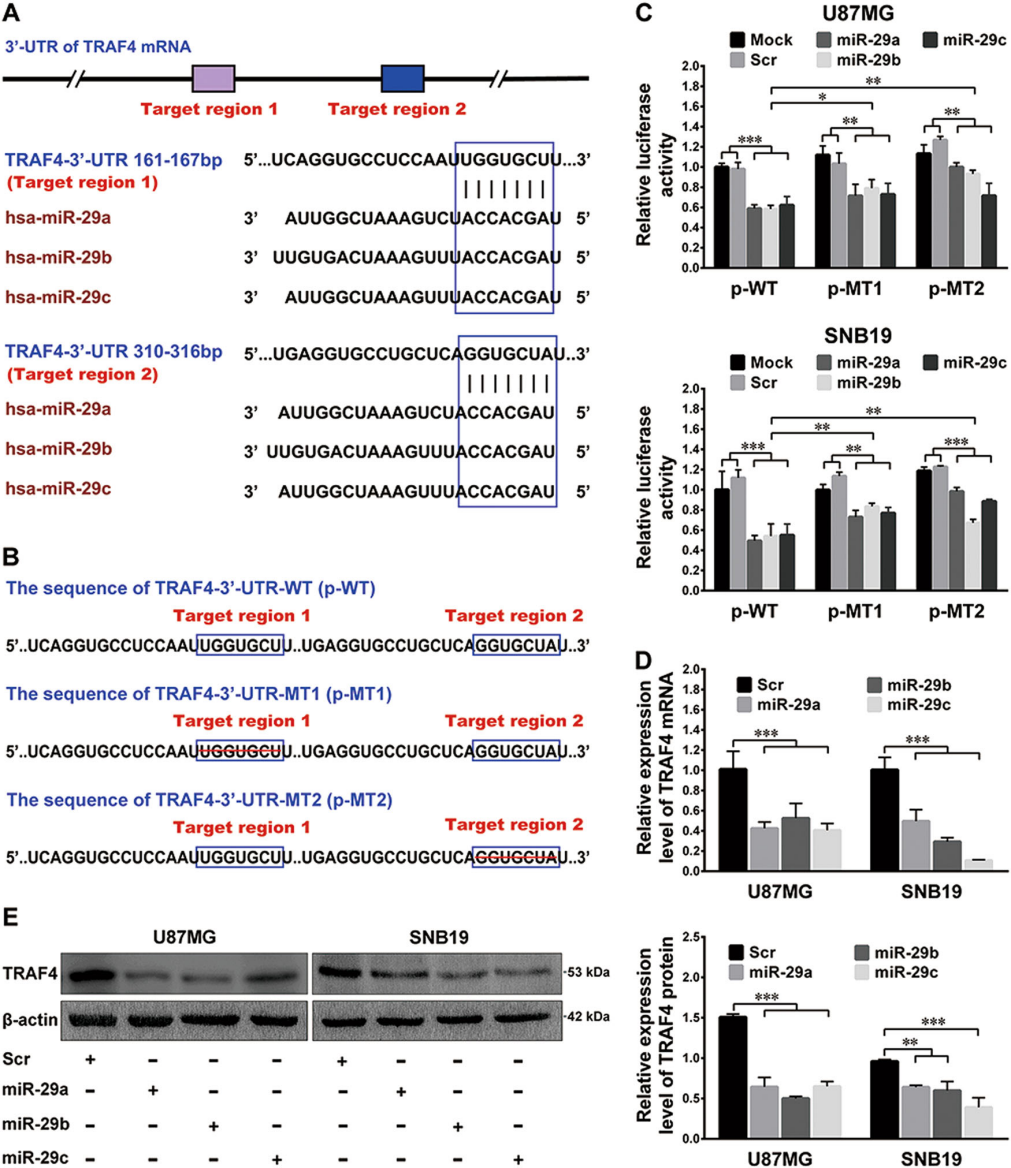
miR-29a/b/c directly target TRAF4 in glioma cells. **a** miR-29a/b/c target regions in the 3′-UTR of TRAF4 mRNA as predicted by the bioinformatics analysis. **b** Schematic illustration of the transcripts of the recombinant luciferase reporter plasmids used in the luciferase assays. The transcript of p-WT (TRAF4-3′-UTR-WT) contains all the target regions for miR-29a/b/c. In the transcripts of p-MT1 and p-MT2 (TRAF4-3′-UTR-MT1 and TRAF4-3′-UTR-MT2), target region 1 or target region 2 was deleted, respectively. **c** Dual-luciferase reporter assays results. U87MG and SNB19 cell lines (Mock) and their Scr and miR-29a/b/c sub-cell lines were transfected with the luciferase reporter plasmid as indicated. **d**, **e** miR-29a/b/c effectively suppressed the expression of TRAF4 mRNA and protein in the miR-29a/b/c sub-cell lines of U87MG and SNB19 cells. All the experiments were performed at least in triplicate and the data in **c**–**e** are presented as the mean ± SD. *P < 0.05; **P < 0.01; ***P < 0.001

For the bioinformatics prediction, two regions in the 3′-UTR of TRAF4 mRNA were predicted to be the putative targets of miR-29a/b/c (Fig. 3a). The silencing effects of the miRNAs were confirmed in U87MG and SNB19 cells by dual-luciferase reporter assays (*P* *<* 0.05–0.001; Fig. 3b, c), which indicated that TRAF4 was a direct target of miR-29a/b/c in glioma cells. In addition, the qRT-PCR and western blot results further verified that miR-29a/b/c significantly decreased the mRNA and protein levels of TRAF4 (*P* < 0.01–0.001; Fig. 3d, e). These data demonstrate that miR-29a/b/c bind directly to the target regions in the 3′-UTR of TRAF4 mRNA and inhibit the expression of TRAF4 via the induction of the degradation of its mRNA.

### TRAF4 overexpression is correlated with miR-29a/b/c down-regulation and predicts a poorer prognosis

**Fig. 4:**
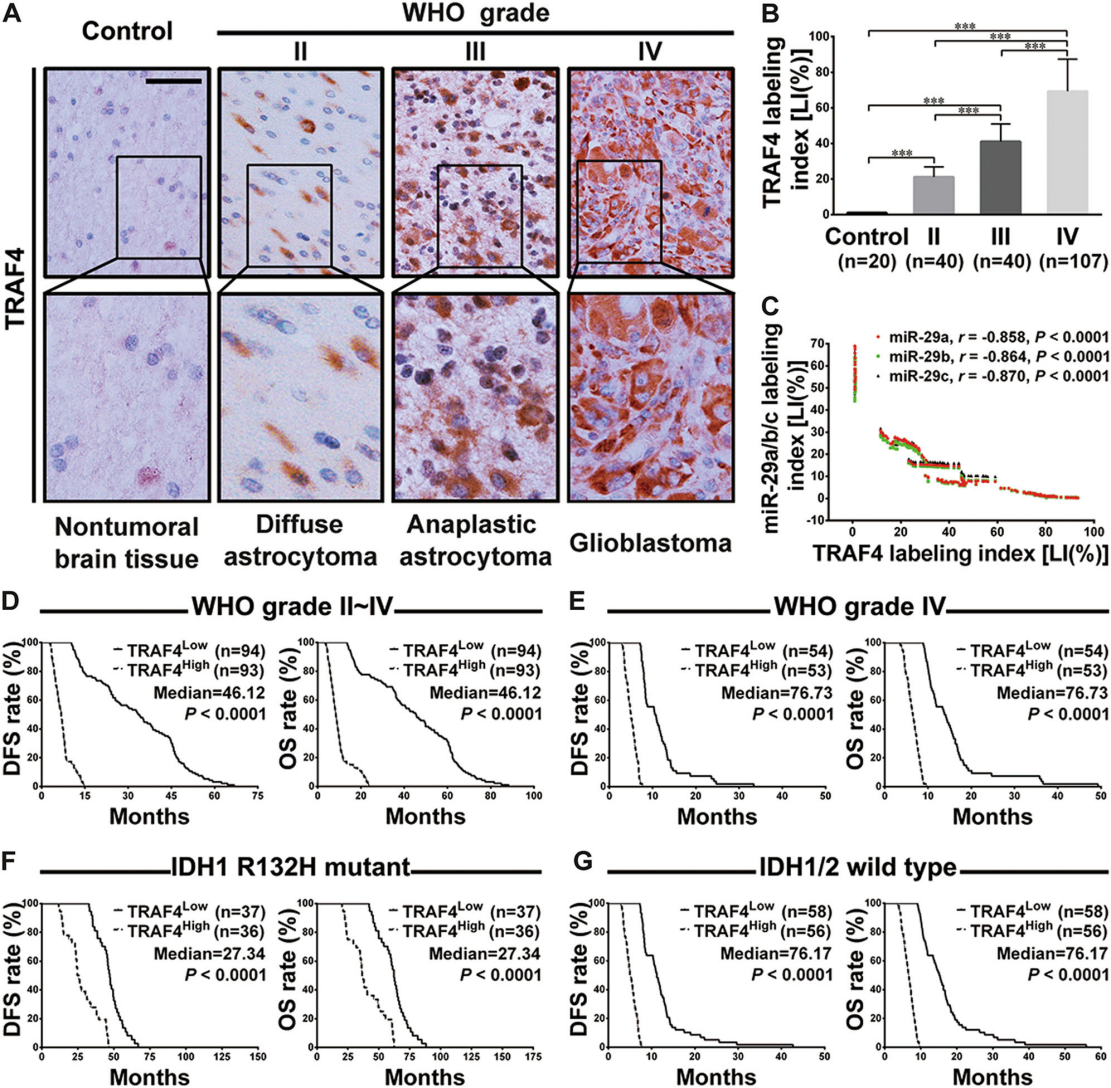
TRAF4 expression is associated with glioma grade, miR-29a/b/c levels and patient prognoses. **a** Representative images of TRAF4 immunohistochemistry. Scale bar: 50 μm. **b** Comparisons of the TRAF4 expression level [labeling index, LI (%)] among the control and the glioma groups. The LI (%) was calculated as described in Fig. 1, and the data are presented as the mean ± SD. ***P *<* 0.001. **c** The LI of TRAF4 was inversely correlated with the LIs of miR-29a/b/c as shown by Pearson analyses. **d–g** Kaplan–Meier survival analyses of the associations between the TRAF4 level and the DFS (left) or OS (right) of the patients. The analyses were conducted in the cohort of all glioma patients (**d**), patients with grade IV gliomas (**e**), patients with IDH1 R132H mutant gliomas (**f**), and patients with IDH1/2 wild-type gliomas (**g**). Patients were stratified into high and low expression subgroups using the medians of the TRAF4 LI of the corresponding cohort

We then sought to detect the endogenous TRAF4 levels in the above glioma specimens and control brain tissues. The IHC results showed that the TRAF4 level was higher in gliomas than in the controls (*P* *<* 0.001) and continued to increase as the glioma grade increased (*P* *<* 0.001; Fig. 4a, b). This result was further verified by the four published datasets from the Oncomine database (*P* < 0.0001; Supplementary Fig. 6d). Moreover, in our glioma specimens, the TRAF4 level was negatively correlated with the miR-29a/b/c levels (miR-29a: *r* = −0.858; miR-29b: *r* = −0.864; miR-29c: *r* = −0.870; *P* < 0.0001; Fig. 4c). Accordingly, the increase in the TRAF4 level was positively associated with the wild-type *IDH1/2* gene, older age, advanced tumor grade, and higher Ki-67 LI (*P* *<* 0.0001; Table 1 and Supplementary Fig. 1c). Kaplan–Meier analyses demonstrated that a higher TRAF4 level predicted a shorter DFS (*P* *<* 0.0001) and OS (*P* *<* 0.0001; Fig. 4d) of our glioma patients. Even within the cohort with similar clinicopathologic features, including tumor grade (Fig. 4e and Supplementary Fig. 6a), *IDH* gene status (Fig. 4f, g), age, and KPS scores (Supplementary Fig. 6b, c), stratification of the patients according to TRAF4 level could still perfectly reflect the differences in outcomes, i.e., the higher the TRAF4 level, the shorter the OS and DFS of the patients (DFS: *P* *<* 0.0001; OS: *P* *<* 0.0001). The prognostic value of TRAF4 in gliomas was further validated by data from TCGA database (OS: *P* < 0.0001; DFS: *P* < 0.01; Supplementary Fig. 6e). These data indicate that the abnormal decrease in miR-29a/b/c levels are important causes of TRAF4 overexpression in gliomas and imply the potential value of TRAF4 in the prognosis of patients with gliomas.

### miR-29a/b/c promote glioma cell apoptosis by blocking the TRAF4/AKT/MDM2/p53 pathway

**Fig. 5:**
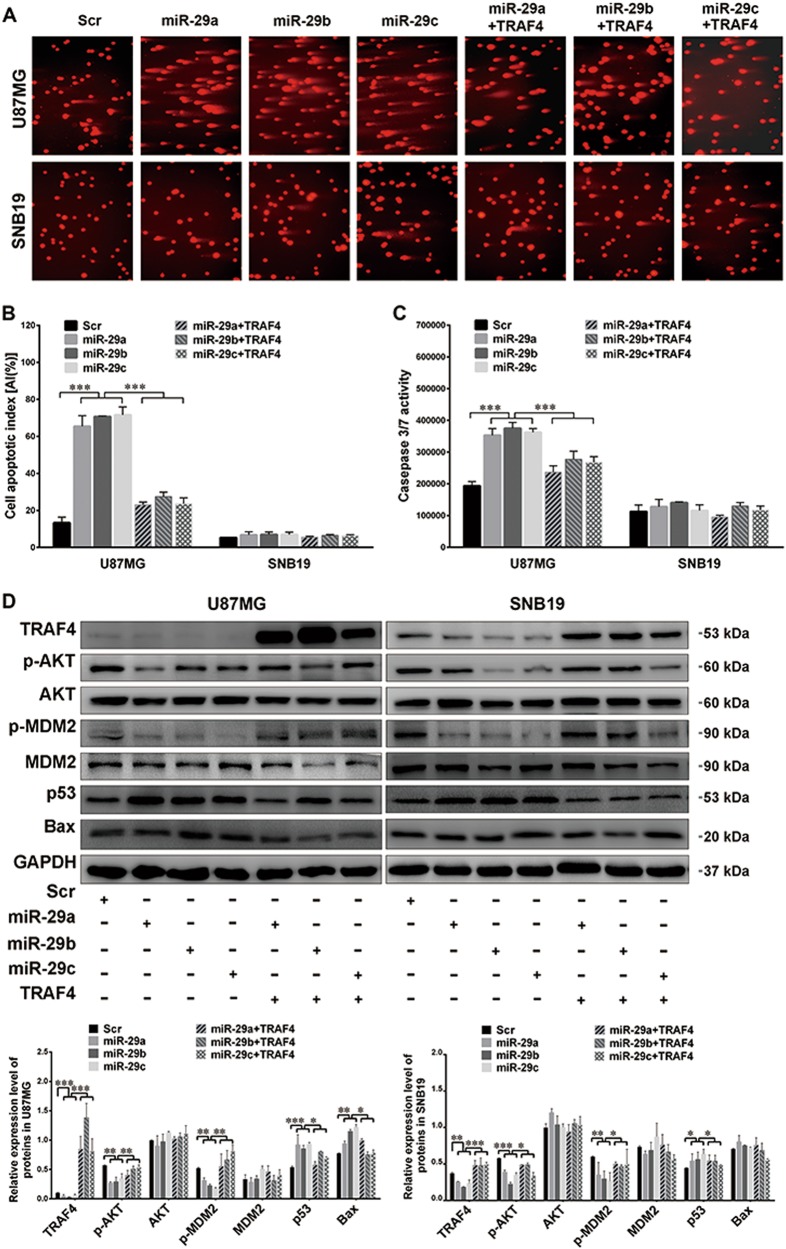
miR-29a/b/c promote glioma cell apoptosis through the TRAF4/AKT/MDM2/p53 pathway. **a, b** Representative images of SCGE (**a**) and the comparison of AIs (**b**). Apoptosis was detected in the Scr and the miR-29a/b/c sub-cell lines of U87MG and SNB19 cells, and the miR-29a/b/c-overexpressing cells transfected with the TRAF4 expression plasmid. **c** Results of the caspase 3/7 activity assay of the cells as indicated. **d** The levels of TRAF4, p-AKT, AKT, p-MDM2, MDM2, p53, and Bax in the extracts of the above cells were detected by western blot using GAPDH as the internal control. All the experiments were performed at least in triplicate and the data in **b**–**d** are presented as the mean ± SD. *P < 0.05; **P < 0.01; ***P < 0.001

To ascertain whether miR-29a/b/c induce cell apoptosis by silencing TRAF4, we transfected the miR-29a/b/c-overexpressing sub-cell lines with the TRAF4-expressing plasmid. The apoptosis detection results showed that although miR-29a/b/c dramatically promoted apoptosis in U87MG and DBTRG-05MG cells, their effects were potentially abrogated by exogenous TRAF4 (*P* *<* 0.001; Fig. 5a–c and Supplementary Fig. 7a, b). However, the apoptotic levels of SNB19 remained constant during miR-29a/b/c overexpression and TRAF4 restoration (*P* *>* 0.05; Fig. 5a–c). To further understand the intracellular signaling network, we focused on p53 and the AKT/MDM2 pathway. Western blotting confirmed that miR-29a/b/c significantly reduced the levels of phosphorylated AKT (p-AKT) and phosphorylated MDM2 (p-MDM2) and increased the levels of p53 in U87MG and SNB19 cells, while TRAF4 overexpression effectively reversed the changes mentioned above (*P* *<* 0.05–0.001; Fig. 5d). However, the total AKT and MDM2 levels were exempted from the impacts of both miR-29a/b/c and TRAF4. Most importantly, the expression of Bax was significantly increased by miR-29a/b/c, and returned when TRAF4 was overexpressed in U87MG cells with the wild-type *TP53* gene (*P* *<* 0.05–0.01; Fig. 5d), but not in SNB19 with mutant *TP53* (Fig. 5d). Overall, these results demonstrate that miR-29a/b/c markedly induce the apoptosis of glioma cells in a p53-dependent manner.

### miR-29a/b/c restrain glioma cell proliferation through the TRAF4/AKT/GSK-3β pathway

**Fig. 6:**
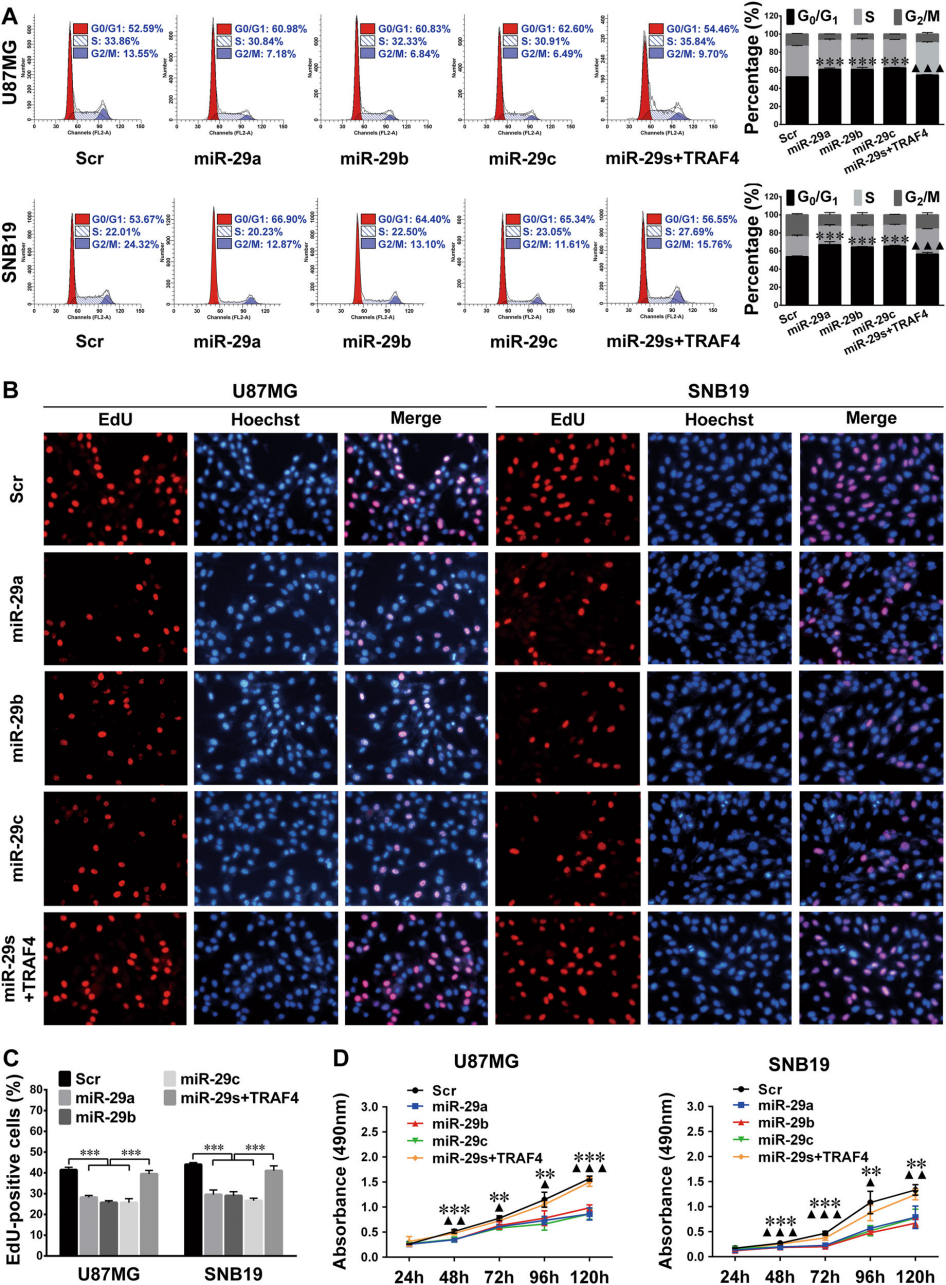
miR-29a/b/c block cell cycle progression and glioma cell proliferation by targeting TRAF4. **a** The representative FCM cell cycle results (left) and the percentages of cells in each phase (right). The percentages of cells in each phase were compared among the Scr and miR-29a/b/c sub-cell lines and the mixture of miR-29a/b/c-overexpressing cells transfected with the TRAF4 expression plasmid (miR-29s + TRAF4). **b, c** EdU assay results (**b**) and quantitative analyses **c** of the aforementioned cells. The EdU-positive and Hoechst-stained cells were counted as described in Fig. 2. **d** Growth curves of the indicated cells drawn with absorbances obtained in MTS assays. All the experiments were performed at least in triplicate and the data in **a**, **c**, **d** are presented as the mean ± SD. ^▲^*P* < 0.05; ^▲▲/^**P < 0.01; ^▲▲▲/^***P < 0.001. The miR-29a/b/c groups compared with the Scr group (*) or the miR-29s + TRAF4 group (^▲^) in **a**, **d**

**Fig. 7:**
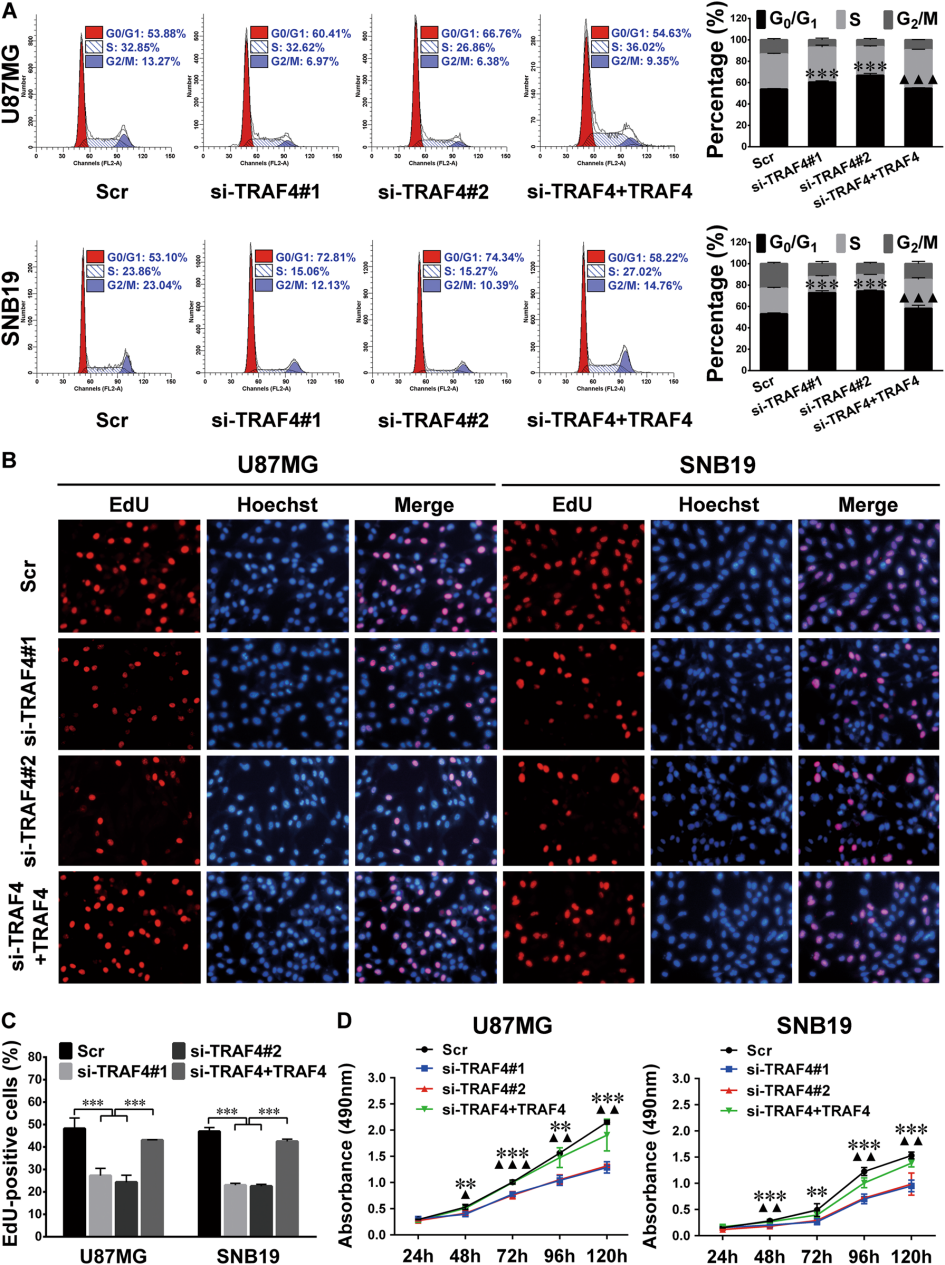
Knockdown of endogenous TRAF4 suppresses cell cycle progression and glioma cell proliferation. **a** FCM cell cycle analysis results. U87MG and SNB19 cells were transfected with a scrambled control sequence (Scr) or TRAF4 siRNAs (si-TRAF4#1 or si-TRAF4#2) or the mixture of TRAF4 siRNAs plus the TRAF4 expression plasmid (si-TRAF4 + TRAF4). **b, c** EdU assay results (**b**) and quantitative analyses (**c**) of the cells as indicated. The EdU-positive and Hoechst-stained cells were counted as described in Fig. 2. **d** Growth curves of the indicated cells. All the experiments were performed at least in triplicate, and the data in **a**, **c**, **d** are presented as the mean ± SD. ^▲^*P* < 0.05; ^▲▲/^**P < 0.01; ^▲▲▲/^***P < 0.001. TRAF4 siRNA groups compared with the Scr group (*) or the si-TRAF4 + TRAF4 group (^▲^) in a, d

To determine whether TRAF4 silencing mediates the adverse effects of miR-29a/b/c on glioma cell proliferation, we transfected the mixture of the miR-29a/b/c-overexpressing sub-cell lines (miR-29s) with the TRAF4-expressing plasmid (miR-29s + TRAF4). The FCM (Fig. 6a), EdU (Fig. 6b, c), and MTS (Fig. 6d) assay results demonstrated that miR-29s dramatically suppressed G1/S-phase transition and tumor cell proliferation, while ectopic TRAF4 efficaciously compromised these effects (*P* < 0.05–0.001). Additionally, transient knockdown of endogenous TRAF4 with specific siRNAs (*P* *<* 0.001; Supplementary Fig. 7c, d) perfectly simulated the suppressive effects of miR-29a/b/c on cell cycle progression and glioma cell proliferation, which were also partially reversed by TRAF4 overexpression (*P* < 0.01–0.001; Fig. 7a–d).

**Fig. 8:**
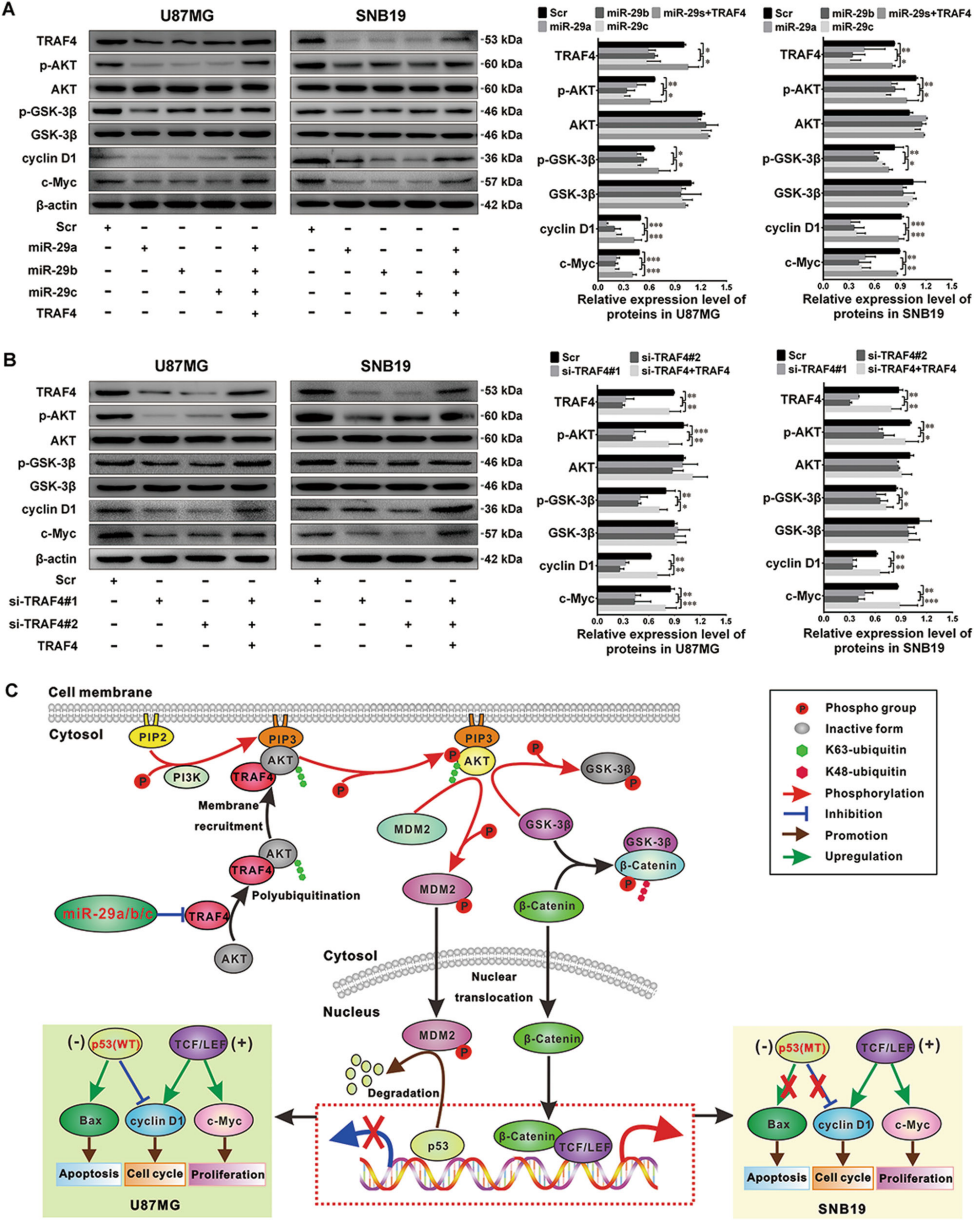
miR-29a/b/c and TRAF4 siRNA suppress the activation of the TRAF4/AKT/GSK-3β pathway. **a, b** Western blot of TRAF4, p-AKT, AKT, p-GSK-3β, GSK-3β, cyclin D1, and c-Myc expression. The Scr and miR-29a/b/c sub-cell lines, the mixture of miR-29a/b/c-overexpressing cells transfected with the TRAF4 expression plasmid, the U87MG and SNB19 cells transfected with Scr or TRAF4 siRNAs (si-TRAF4#1 or si-TRAF4#2) or the mixture of TRAF4 siRNAs plus the TRAF4 expression plasmid were homogenized, and the total protein was extracted and subjected to western blot detection. All the experiments were performed at least in triplicate and the data in **a**, **b** are presented as the mean ± SD. *P < 0.05; **P < 0.01; ***P < 0.001. **c** Schematic illustration of the molecular pathways underlying the anti-glioma effects of miR-29a/b/c

To further explore the pathway through which miR-29a/b/c suppress glioma cell proliferation, we focused on the crucial effectors downstream of TRAF4, including AKT, GSK-3β, c-Myc, and the G1/S-phase checkpoint regulator cyclin D1. As shown in Fig. 8a, b, the levels of TRAF4, p-AKT, phosphorylated GSK-3β (p-GSK-3β), c-Myc, and cyclin D1 were significantly reduced in U87MG and SNB19 cells that overexpressed miR-29a/b/c or that were transfected with TRAF4 siRNAs (*P* *<* 0.05–0.001). In contrast, the total AKT and GSK-3β levels remained constant among all the groups. Moreover, TRAF4 overexpression reversed the above-mentioned changes in protein levels induced by miR-29a/b/c restoration and TRAF4 knockdown (*P* *<* 0.05–0.001). These results indicate that miR-29a/b/c inhibit G1/S-phase transition and glioma cell proliferation by direct targeting of the TRAF4/AKT/GSK-3β pathway and the suppression of c-Myc and cyclin D1 expression (Fig. 8c).

## Discussion

The miR-29 family members have been recognized as important tumor suppressors in various malignant tumors^13–17,20^. However, their anti-tumor roles and clinical relevance remain to be investigated in a large cohort of glioma patients. In the present study, we demonstrated that miR-29a/b/c effectively induced apoptosis and inhibited the proliferation of glioma cells by targeting TRAF4, and thereby we validated the miR-29 members as important glioma suppressors. We also demonstrated that these suppressive miRNAs were expressed at low levels in gliomas, especially in high-grade gliomas and that their levels were closely associated with patient outcomes. These findings not only enrich our knowledge of the crucial molecular events during glioma formation, but they also imply the usefulness of these miRNAs as prognostic biomarkers for glioma patients.

A comprehensive study of miRNAs and their regulatory networks is a prerequisite for the scientific evaluation of their practical values in the molecular subclassification, diagnosis, and prognosis of gliomas^6–9^. In this study, we verified that the expression levels of miR-29a/b/c and TRAF4 were closely correlated with tumor grade and proliferation index, which suggests that they can be used as accessory indicators for glioma grading. A survival analysis showed that even within the current frame of prognostic evaluation (including pathological grade, IDH status, age and KPS), the expression levels of miR-29a/b/c and TRAF4 could still provide us with additional reliable predictive information on patient outcomes and could improve the accuracy of the prognostic assessment. In our pathologic analysis, we also found that the Ki-67 index, a widely accepted scale for proliferation, was not only increased as the glioma grade increased but also inversely correlated with miR-29a/b/c levels and was positively correlated with the TRAF4 level. This strongly indicates that miR-29a/b/c and TRAF4 are important regulators of glioma cell proliferation. Moreover, the inverse correlations between the levels of miR-29a/b/c and TRAF4 imply that the abnormal decrease in miR-29s is responsible for the overexpression of TRAF4 in gliomas. This conclusion was further supported by the luciferase assay results which confirmed that TRAF4 was a natural target of miR-29a/b/c in glioma cells.

Apoptotic inhibition and vigorous proliferation of tumor cells are not only the hallmarks of malignant gliomas, but they are also important cause of their rapid growth^3,31^. Our in vitro results showed that all the miR-29 members efficiently inhibited the proliferation of glioma cells and that they also induced apoptosis in certain glioma cell lines such as U87MG and DBTRG-05MG. Furthermore, since the above-mentioned anti-glioma effects of miR-29a/b/c could be simulated perfectly by the specific siRNAs of TRAF4 and could be partially reversed by TRAF4 overexpression, we conclude that miR-29a/b/c suppress glioma growth at least partly by silencing TRAF4. These results highlight the importance of miR-29a/b/c as tumor suppressors to combat malignant gliomas.

TRAF4 has recently been found to promote tumorigenesis through the activation of the central signaling node AKT^32,33^, which is one of the most pivotal and versatile protein serine/threonine kinases at the core of glioma pathogenesis^34,35^. TRAF4 catalyzes the synthesis of the K63-linked polyubiquitin chain which recruits AKT to the cell membrane, where AKT is phosphorylated and activated^32^. Upon activation, AKT phosphorylates MDM2 and facilitates its translocation to cell nuclei. In cell nuclei, MDM2 induces the ubiquitination and degradation of both wild type and mutant p53 (refs. ^27,36–39^) and thereby inhibits the transcription of *Bax* and cell apoptosis. In addition, GSK-3β is a critical component of the β-catenin destruction complex, as it captures, phosphorylates, and ubiquitinates cytoplasmic β-catenin. p-AKT catalyzes the inactivation of phosphorylated GSK-3β, and thereby prevents β-catenin from proteasome mediated degradation^40–42^. Free β-catenin then translocates to cell nuclei where it activates the transcription of *c-Myc* and *cyclin D1* by forming a complex with the transcription factor TCF/LET; this in turn accelerates G1/S-phase transition and cell proliferation^40,43^.

In the present study, although miR-29s-induced TRAF4 knockdown managed to block MDM2 phosphorylation and p53 degradation in both U87MG and SNB19 cell lines, it induced Bax expression and apoptosis only in U87MG with wild-type *TP53*. However, in SNB19 cells, since mutant p53 could not activate transcription of the *Bax* gene, miR-29a/b/c failed to induce apoptosis in these cells. The DBTRG-05MG (*TP53* wild-type) and U251 (*TP53* mutant) cell lines were shown to be sensitive and resistant to miR-29s induced apoptosis, respectively, which was consistent with the findings discussed above. These results demonstrate that the pro-apoptotic effects of miR-29a/b/c require the presence of wild-type p53 and that miR-29a/b/c induce the apoptosis of glioma cells via the AKT/MDM2/p53 pathway in a p53-dependent manner (Fig. 8c). Moreover, we found that miR-29s-induced TRAF4 knockdown markedly blocked the phosphorylation of AKT and GSK-3β as well as the expression of cyclin D1 and c-Myc in glioma cells. Based on these findings, we conclude that miR-29a/b/c suppress G1/S-phase transition and the proliferation of glioma cells by targeting TRAF4 and down-modulating the AKT/GSK-3β pathway (Fig. 8c). These conclusions were also supported by siRNA simulation and TRAF4 rescue experiments.

In summary, our study indicates that miR-29a/b/c promote apoptosis and inhibit the proliferation of glioma cells by the direct targeting of TRAF4. The abnormal decrease in miR-29a/b/c is an important cause of TRAF4 overexpression and plays crucial roles in the tumorigenesis and malignant progression of gliomas. Our results underscore the practical values of these molecules as novel biomarkers for glioma grading and prognostic assessment, and imply that miR-29a/b/c and TRAF4 can be used as therapeutic candidates and as a target for malignant glioma, respectively.

### Bullet Points

The expression of miR-29s decreases as the glioma grade increases and predicts worse patient prognosis.

Abnormal decreases in miR-29s are important factors that lead to TRAF4 overexpression

miR-29s induce glioma cell apoptosis in a p53-dependent manner through the TRAF4/AKT/MDM2 pathway.

miR-29s restrain cell proliferation by directly targeting the TRAF4/AKT/GSK-3β pathway.

miR-29s and TRAF4 are potential biomarkers for glioma grading and prognosis.

## Electronic supplementary material

Supplementary information
