# Supplementary material for: miR-29s function as tumor suppressors in gliomas by targeting TRAF4 and predict patient prognosis
Source: Cell Death Dis. 2018 Oct 22;9(11):1078. doi: 10.1038/s41419-018-1092-x (PMC6197255; doi:10.1038/s41419-018-1092-x)
Supplement: Supplementary file 1 — Supplementary information [file 41419_2018_1092_MOESM1_ESM.pdf]

1. **Supplementary figures and figure legends for supplementary figures 1-7.**
2. **Supplementary tables for supplementary tables 1-5.**

Supplementary Figures and Figure Legends

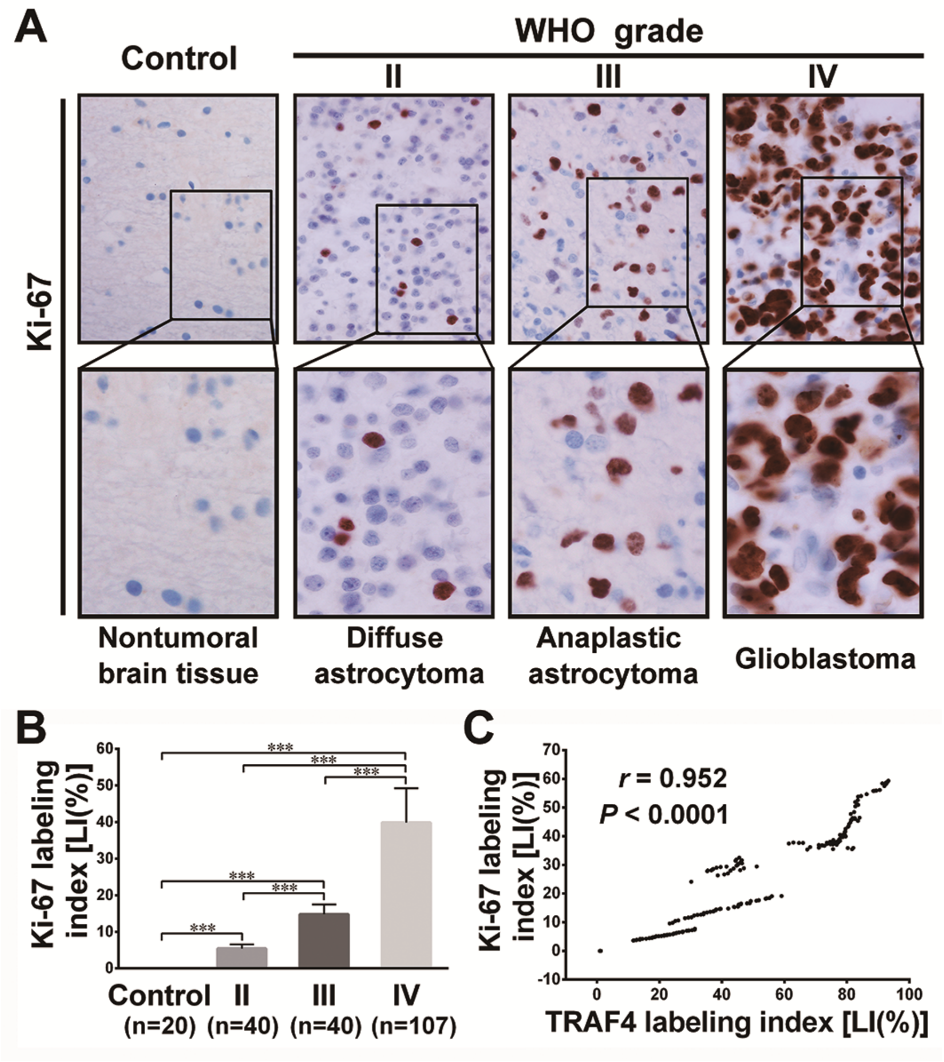

Supplementary Fig. 1. Ki-67 index is correlated with glioma grade and TRAF4 level. (**a**) Representative images of Ki-67 IHC detection. Scale bar: 50 μm. (**b**) Comparisons of the Ki-67 index among the control and the glioma groups. Data in b are presented as the mean ± SD. *** $P < 0.001$. (**c**) Pearson correlation analysis shows that the TRAF4 LI is positively correlated with the Ki-67 index in gliomas.

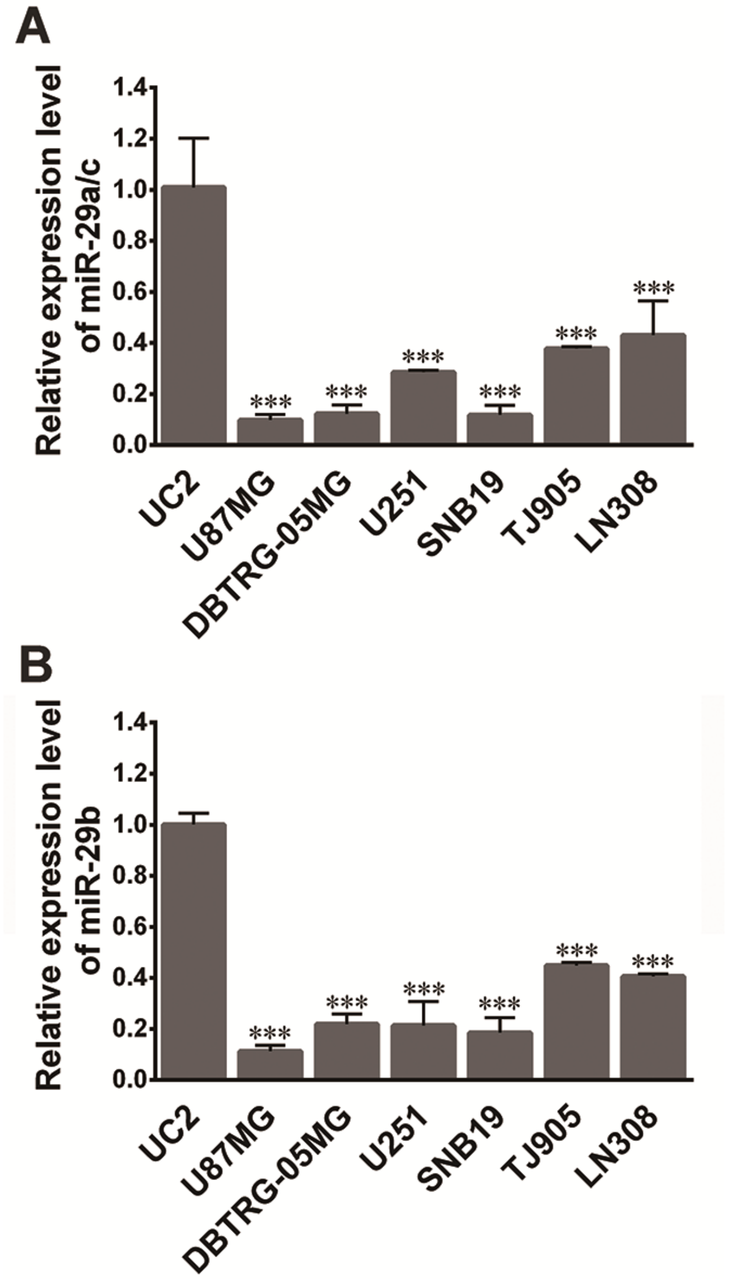

Supplementary Fig. 2. miR-29a/b/c expression levels in UC2 and six glioblastoma cell lines. The endogenous miR-29a/c (a) and miR-29b (b) levels in the indicated cell lines were measured by qRT-PCR and normalized against the corresponding levels of U6. The relative levels of miR-29a/c and miR-29b in immortalized human astrocyte cell line UC2 were arbitrarily set to 1.0. All the measurements were repeated at least three times, and the data are presented as the mean ± SD; *** $P<0.001$.

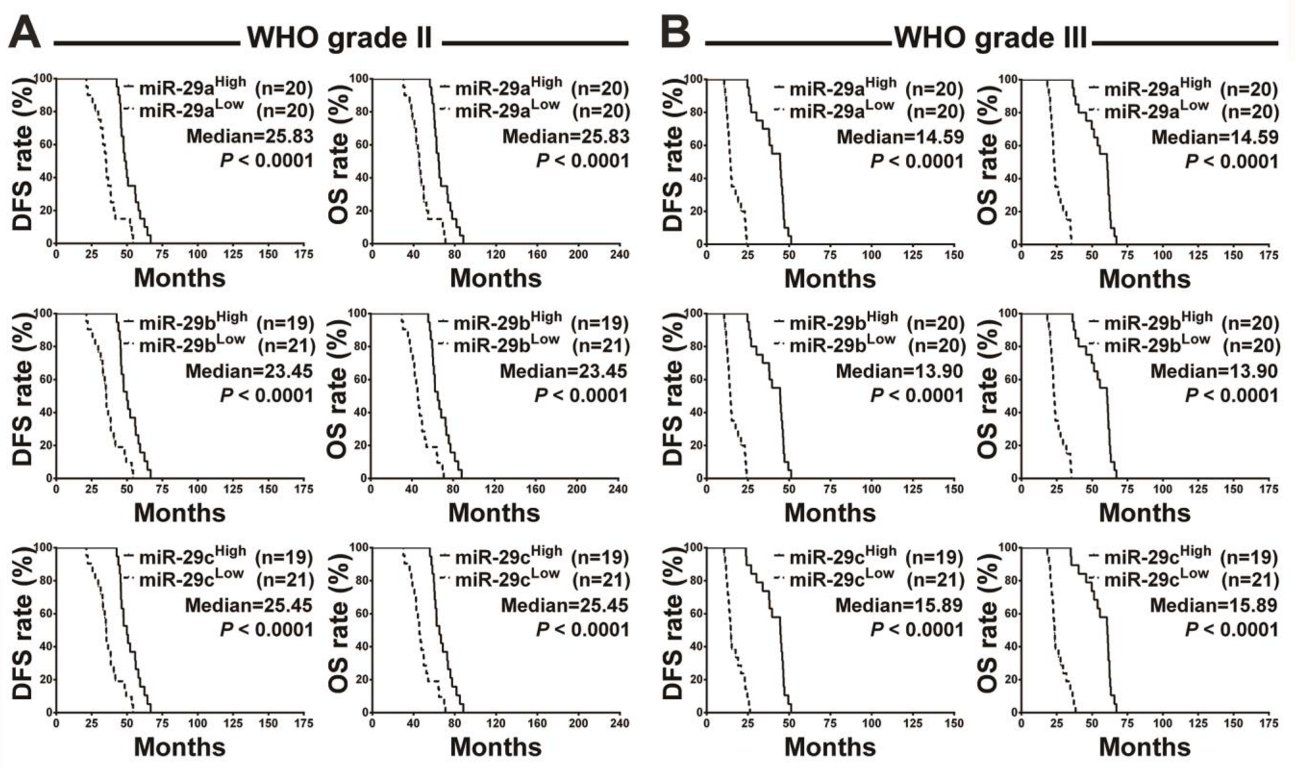

Supplementary Fig. 3. miR-29a/b/c levels are associated with the survival of the patients with WHO grade II or grade III gliomas. The DFS (left) and OS (right) of the patients with WHO grade II (a) or grade III (b) gliomas were analyzed by the Kaplan-Meier method. Patients were stratified into the high and low expression subgroups using the medians of miR-29a/b/c LIs of the corresponding cohort.

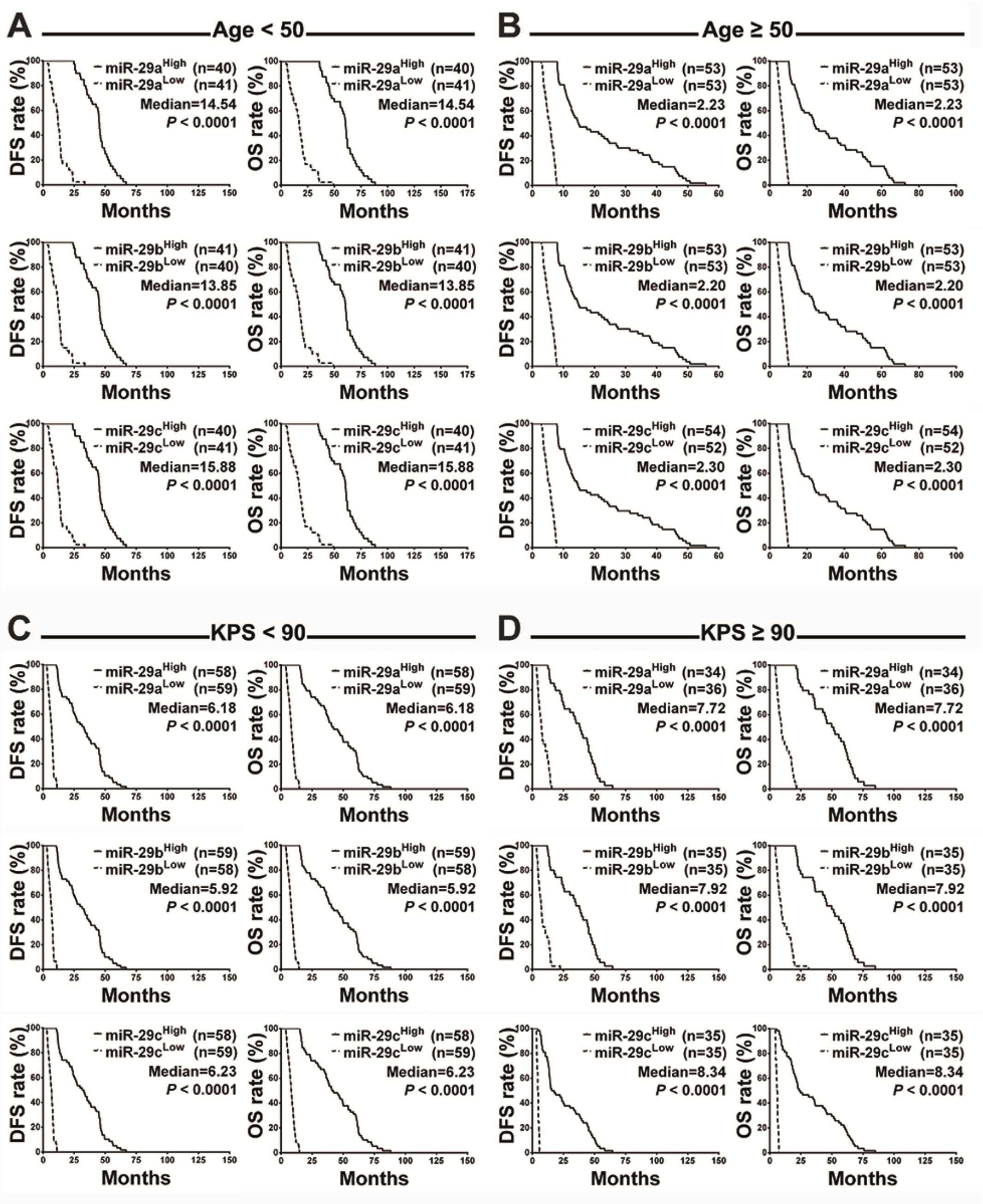

Supplementary Fig. 4. miR-29a/b/c levels are correlate with the prognoses of glioma patients in the same age and KPS groups. Patients were divided into the age < 50 (a), age ≥ 50 (b), KPS < 90 (c) and KPS ≥ 90 (d) groups according to their clinical features. The DFS (left) and OS (right) of the patients were analyzed by the Kaplan-Meier method. Patients were further stratified into high and low expression subgroups using the medians of miR-29a/b/c LIs of the corresponding groups.

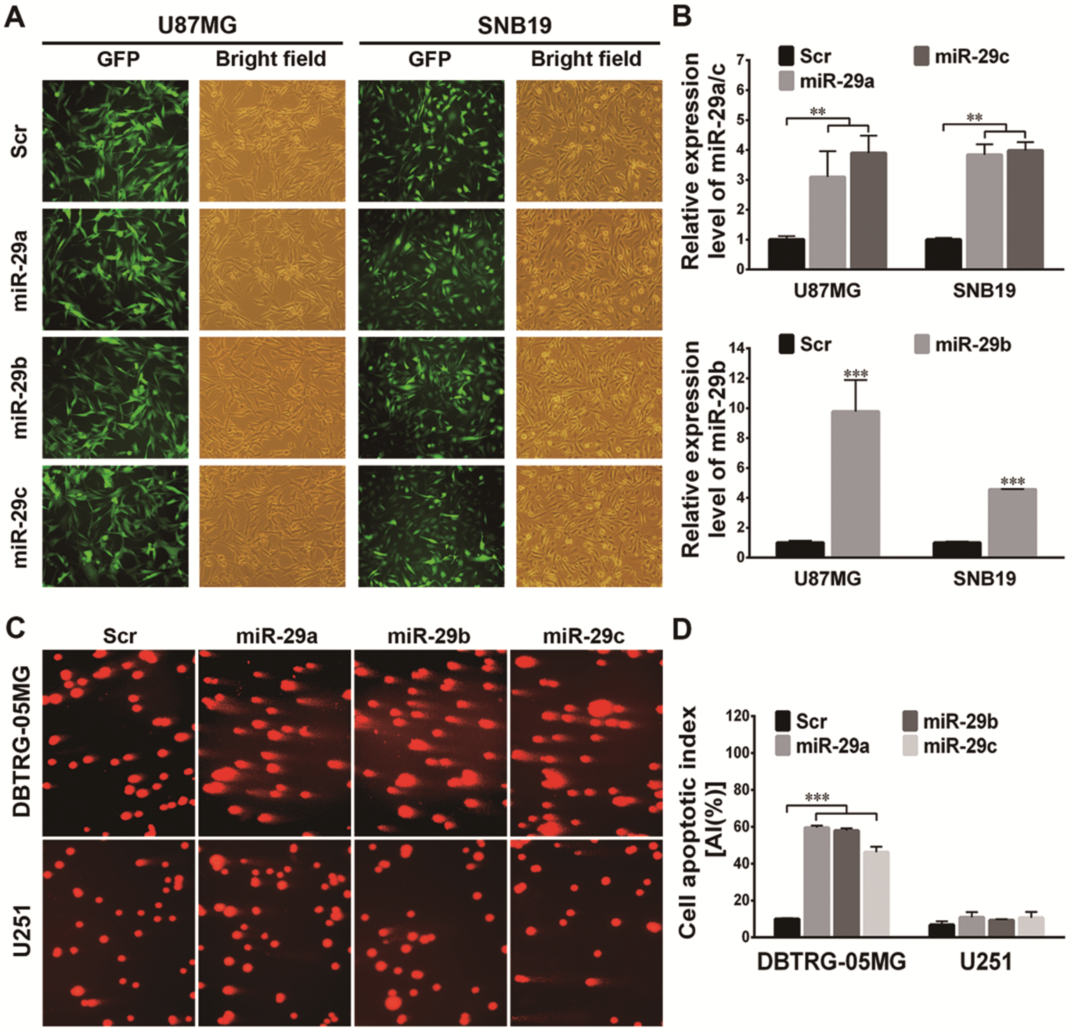

Supplementary Fig. 5. The infection and expression efficiencies of miR-29a/b/c lentivirus in glioma cells and the pro-apoptotic effects of miR-29a/b/c. (**a**) The validities of the Scr and miR-29a/b/c sub-cell lines of U87MG and SNB19 were confirmed by the GFP reporter. (**b**) The expression levels of miR-29a/c and miR-29b in the Scr and miR-29a/b/c sub-cell lines were measured by qRT-PCR using U6 as the internal control. The relative levels of the Scr sub-cell lines were set to 1.0. (**c, d**) Apoptosis was detected by SCGE (c) and the AIs (%) were compared among the miR-29a/b/c overexpressing groups and the Scr group (d) in DBTRG-05MG and U251 cells. All the experiments were performed in triplicate and the data are presented as the mean ± SD. ** $P < 0.01$; *** $P < 0.001$.

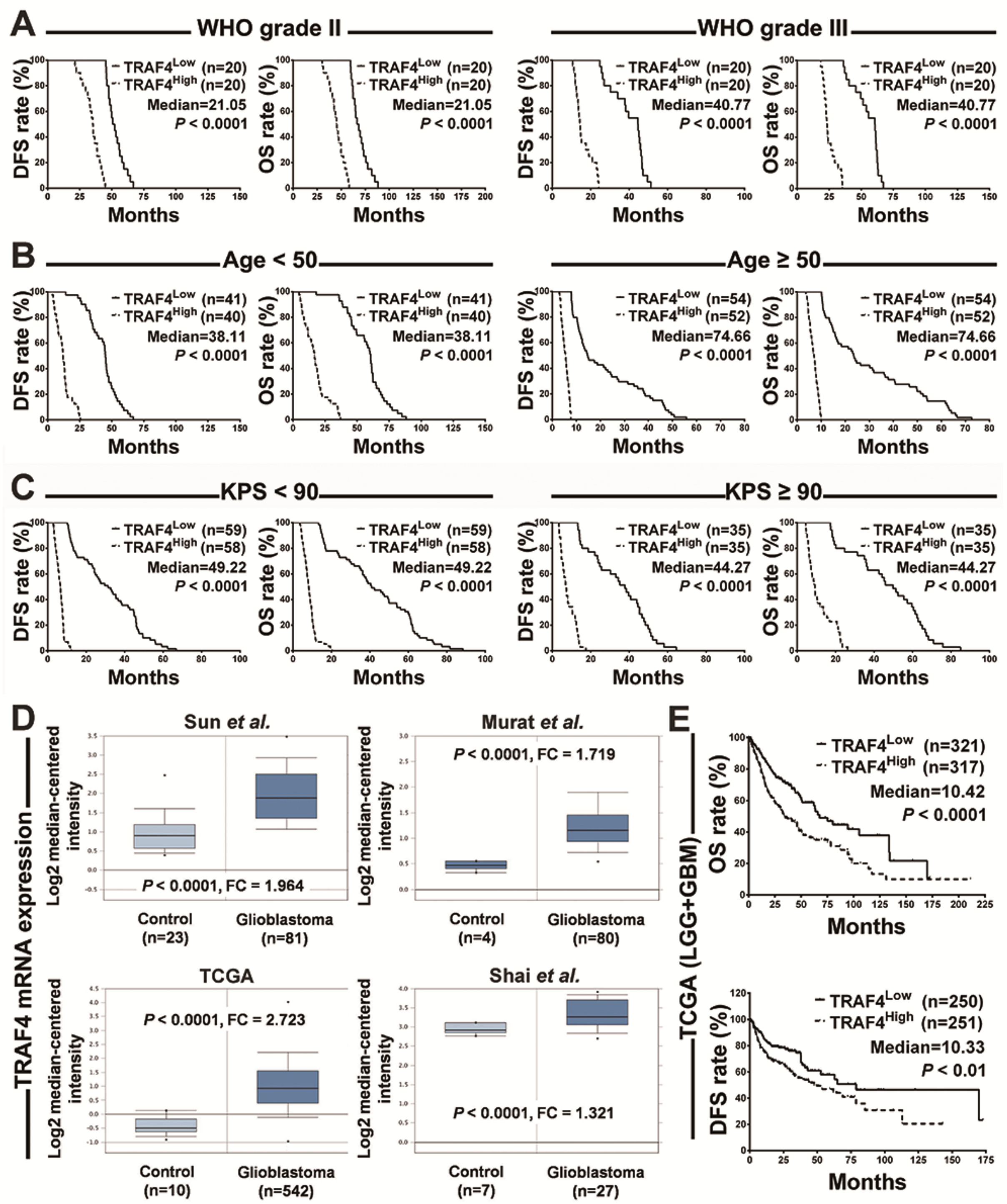

Supplementary Fig. 6. TRAF4 expression is correlated with the prognoses of glioma patients. (**a, b, c**) Kaplan-Meier analyses of the DFS (left) and OS (right) of the patients. Patients were grouped according to glioma grade (a), onset age (b) and KPS scores (c), and further stratified into high and low expression subgroups with the medians of TRAF4 LI of

the corresponding groups. (**d**) Comparison of TRAF4 mRNA levels between control brain tissues and glioblastoma tissues using the data from 4 published datasets. Sun *et al.*: control (n=23), glioblastoma (n=81); TCGA: control (n=10), glioblastoma (n=542); Murat *et al.*: control (n=4), glioblastoma (n=80); Shai *et al.*: control (n=7), glioblastoma (n=27). (**e**) Kaplan-Meier analysis of the OS (n=638) and DFS (n=501) of the lower grade glioma (LGG) patients and the glioblastoma (GBM) patients. Data were obtained from TCGA database. Patients were stratified into high and low expression subgroups using the medians of the relative TRAF4 levels as indicated.

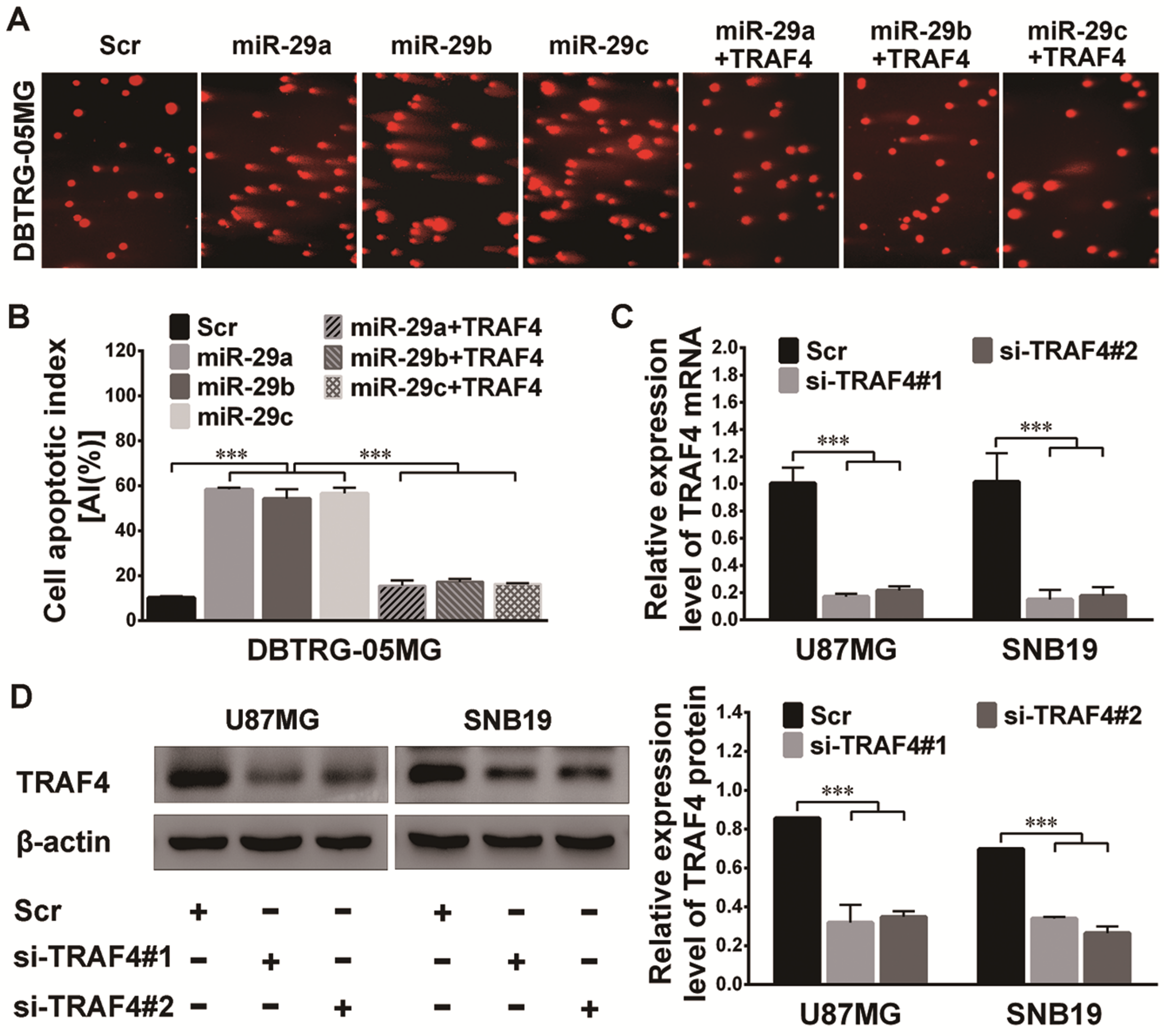

Supplementary Fig. 7. miR-29a/b/c promote the apoptosis of glioma cells by targeting TRAF4 and the knockdown efficiencies of TRAF4 siRNAs in glioma cells. (**a**) Apoptotic cells were detected in the Scr and miR-29a/b/c sub-cell lines of DBTRG-05MG and the miR-29a/b/c overexpressing cells transfected with TRAF4 expression plasmid by SCGE. (**b**) Comparison of the AIs (%) among the groups as indicated. (**c, d**) U87MG and SNB19 cells were transfected with either the TRAF4 siRNAs (si-TRAF4#1 or si-TRAF4#2) or a scrambled oligonucleotide (Scr). The expression levels of TRAF4 mRNA and protein were measured by qRT-PCR (c) and Western blot (d), respectively. In qRT-PCR, GAPDH mRNA was used as the internal control, and the relative levels of the Scr group was set to 1.0. In Western blot, β-actin protein was used as the internal control. All experiments were performed at least in triplicate and the data in b, c, d are presented as the mean ± SD. *** $P < 0.001$.

Supplementary tables

Supplementary Table 1. The clinicopathological features of the glioma patients enrolled in this study

Feature	WHO Grade		
	II (n=40)	III (n=40)	IV (n=107)
IDH status			
Mutant type (IDH1 R132H)	35	33	5
Wild-type (IDH1/2)	5	7	102
Gender			
Male	22	22	70
Female	18	18	37
Age (Year, Mean±SD)	42.73±11.88	47.68±15.25	55.64±12.38
Age < 50	29	22	30
Age ≥ 50	11	18	77
KPS			
KPS < 90	23	24	70
KPS ≥ 90	17	16	37
Predominant side			
Left	20	17	58
Right	17	21	46
Middle	3	2	3
Predominant location			
Frontal lobe	30	26	47
Temporal lobe	6	8	40
Parietal lobe	1	3	11
Occipital lobe	1	1	6
Insular lobe	0	0	1
Cerebellum	2	1	1
CPA	0	0	1
Third ventricle	0	1	0

Abbreviation: SD, Standard deviation; KPS, Karnofsky performance score.

Supplementary Table 2. The sequences of the probes for miR-29a/b/c and the scrambled oligonucleotide (Scr) used in ISH

Oligonucleotide probes	Sequence
DIG-labeled LNA miR-29a	5’- TAACCGATTTCAGATGGTGCTA-3’
DIG-labeled LNA miR-29b	5’-AACACTGATTTCAAATGGTGCTA-3’
DIG-labeled LNA miR-29c	5’-TAACCGATTTCAAATGGTGCTA-3’
DIG-labeled LNA Scr	5’-CGTATAGGCCCAAGAATTAGG-3’

Abbreviation: DIG, digoxin; LNA, locked nucleic acid-modified.

Supplementary Table 3. The sequences of TRAF4 siRNAs and the scrambled oligonucleotide

dsRNA oligonucleotides	Sequence
TRAF4 siRNA#1 (si-TRAF4#1)	5'- GCACCUACUGCACUAAGGATT-3'<br>3'- TTCGUGGAUGACGUGAUUCCU-5'
TRAF4 siRNA#2 (si-TRAF4#2)	5'- CCAGGACAUUCGAAAGCGATT -3'<br>3'- TTGGUCCUGUAAGCUUUCGCU -5'
Scrambled control sequence	5'-UUCUCCGAACGUGUCACGUTT-3'<br>3'-TTAAGAGGCUUGCACAGUGCA-5'

Supplementary Table 4. The sequences of the primers used in qRT-PCR detection

Primers	Orientation	Sequence
TRAF4	forward	5’- CATCCACAGTGAGGAGGGCT-3’
	reverse	5’- TTCATGGGGCAGCGATTAG-3’
GAPDH	forward	5’-TGCACCACCAACTGCTTAGC-3’
	reverse	5’-GGCATGGACTGTGGTCATGAG-3’

Supplementary Table 5. Primers used for amplification of TRAF4-3’-UTR-WT, TRAF4-3’-UTR-MT1 and TRAF4-3’-UTR-MT2

Primers	Orientation	Sequence
WT	forward	5’-GCGATCGCACCCCCGTCAGCTGCTTCTG-3’
	reverse	5’-CCGCTCGAGTTTCTCCCTCCCCAATTCCCACC-3’
MT1	forward	5’-TCAGGTGCCTCCAATTTCAGCCCTGGCCCCTGT-3’
	reverse	5’-ACAGGGGCCAGGGCTGAAATTGGAGGCACCTGA-3’
MT2	forward	5’-ACTGAGGTGCCTGCTCAATGTCCCAAGAGCCATAAG-3’
	reverse	5’-CTTATGGCTCTTGGGACATTGAGCAGGCACCTCAGT-3’
